# Carriage of Extended-Spectrum-*β*-Lactamase- and AmpC-*β*-Lactamase-Producing *Enterobacteriaceae* (ESBL-PE) in Healthy Community and Outpatient Department (OPD) Patients in Nepal

**DOI:** 10.1155/2020/5154217

**Published:** 2020-02-11

**Authors:** Dipendra Kumar Mandal, Shiv Kumar Sah, Shyam Kumar Mishra, Sangita Sharma, Hari Prasad Kattel, Sanjeet Pandit, Pranav Kumar Yadav, Ujjwal Laghu, Rajani Lama, Niranjan Prasad Sah, Jeevan Bahadur Sherchand, Keshav Parajuli, Anup Bastola, Sher Bahadur Pun, Basista Prasad Rijal, Bharat Mani Pokharel

**Affiliations:** ^1^Department of Laboratory Medicine, Manmohan Memorial Institute of Health Sciences, Kathmandu, Nepal; ^2^Sukraraj Tropical and Infectious Diseases Hospital, Teku, Kathmandu, Nepal; ^3^Faculty of Pharmaceutical Science, Purbanchal University, Little Buddha College of Health Science, Minbhawan, Kathmandu, Nepal; ^4^Department of Microbiology, Tribhuvan University Teaching Hospital, Kathmandu, Nepal; ^5^National Public Health Laboratory, HIV Reference Unit, Kathmandu, Nepal; ^6^Department of Laboratory Medicine, Udaypur District Hospital, Udaypur, Nepal; ^7^Department of Microbiology, Grande International Hospital, Kathmandu, Nepal

## Abstract

**Background:**

Extended-spectrum *β*-lactamase (ESBL)- and AmpC-*β*-lactamase (ESBL)- and AmpC-*Enterobacteriaceae* have recently emerged as a public threat in the treatment of nosocomial as well as community-acquired infections. Very little information is currently available about its existence in Nepal. We, therefore, aim to determine the prevalence of ESBL and AmpC-*β*-lactamase (ESBL)- and AmpC-*Enterobacteriaceae* have recently emerged as a public threat in the treatment of nosocomial as well as community-acquired infections. Very little information is currently available about its existence in Nepal. We, therefore, aim to determine the prevalence of ESBL and AmpC-

**Methods:**

During a 6-month period (November 2014–April 2015), a total of 190 stool specimens from 190 participants were obtained from different population. Of the total 260 fecal isolates, 152 from outpatient department (OPD) and 108 from healthy volunteer were collected. Stool specimens were cultured and enterobacterial isolates were subjected to antimicrobial susceptibility tests according to the standard microbiologic guidelines. ESBL was screened using ceftazidime (CAZ, 30 *μ*g) and cefotaxime (CTX, 30 *μ*g) and cefotaxime (CTX, 30 *β*-lactamase (ESBL)- and AmpC-

**Results:**

The prevalence of ESBL, AmpC-*β*-lactamase (ESBL)- and AmpC-*β*-lactamase (ESBL)- and AmpC-*Enterobacteriaceae* have recently emerged as a public threat in the treatment of nosocomial as well as community-acquired infections. Very little information is currently available about its existence in Nepal. We, therefore, aim to determine the prevalence of ESBL and AmpC-*E. coli* was 70.2% followed by *K. pneumoniae* (12.7%), and among AmpC-*β*-lactamase (ESBL)- and AmpC-*E. coli* was 70.2% followed by *E. coli* was 70.2% followed by *K. pneumoniae* (12.7%), and among AmpC-*K. pneumoniae* (12.7%), and among AmpC-*C. freundii* 2/7 (28.57%) were detected highest among AmpC-*β*-lactamase (ESBL)- and AmpC-

**Conclusion:**

Our study revealed a high prevalence of ESBL- and AmpC-*β*-lactamase-producing enteric pathogen in Nepalese OPD and healthy population. The significant increase of these isolates and increased rate of drug resistance indicates a serious threat that stress the need to implement the surveillance system and a proper control measure so as to limit the spread of ESBL-producing *Enterobacteriaceae* (ESBL-PE) in both OPD as well as in community. Therefore, healthcare providers need to be aware that ESBL- and AmpC-*β*-lactamase-producing strains are not only circulating in hospital environments but also in the community and should be dealt with accordingly.*β*-lactamase (ESBL)- and AmpC-*Enterobacteriaceae* have recently emerged as a public threat in the treatment of nosocomial as well as community-acquired infections. Very little information is currently available about its existence in Nepal. We, therefore, aim to determine the prevalence of ESBL and AmpC-*β*-lactamase (ESBL)- and AmpC-

## 1. Introduction

ESBL-producing *Enterobacteriaceae* (ESBL-PE), especially *E. coli* and *K. pneumonia*, have recently emerged as a major public health threat [1, 2] and are associated with high mortality, increased length of hospital stay, and high cost [3–5]. On the other hand, multidrug resistance, secondary to widespread use of antibiotic, is increasingly seen in these isolates. The bacterial strain producing extended-spectrum beta-lactamase (ESBL) and AmpC-*β*-lactamase enzymes, capable of hydrolyzing penicillins, monobactams, and 3rd-generation cephalosporins but not cephamycin and carbapenem, is being abundantly found in *Enterobacteriaceae* [6, 7]. The other mechanism of drug resistance encompasses the large plasmids that carry the beta-lactamase genes which confer resistance to other antibiotic classes as well, leaving very few treatment option and increased risk of treatment failure in patients infected with such strains. Until recently, most infections caused by ESBL-producing *E. coli* were hospital-acquired. BlaCTX-M genes originate from environmental bacteria but have migrated to highly transmissible plasmids, which have been linked to ESBL circulation in the community. The community can thus represent a reservoir for ESBLs not yet detected in clinical isolates [8]. Though ESBL initially associated with nosocomial outbreak, several reports have recently revealed more complex situation, with a dramatically increase in community isolates in many parts of the world [9–12]. The actual prevalence of ESBL-producing enterobacterial strain varies, depending on nature of the studies, participants involved, and geographical region. Reports from the Western Pacific, Eastern Mediterranean, and Southeast Asia regions showed the highest carriage rates and the most alarming being the recent ascending trends [13–15]. Despite these growing worrisome issues, the data regarding prevalence and resistance mechanism of ESBL-producing *Enterobacteriaceae* in hospital as well as community setting in Nepal are lacking. Therefore, the present study aimed to determine the carriage of ESBL-PE in healthy community as well as OPD participants and further to determine the drug resistance to ESBL-PE isolates.

## 2. Methods

### 2.1. Study Design and Duration

This observational and cross-sectional study was conducted at the Department of Microbiology, Tribhuvan University Teaching Hospital (TUTH), over a period of six months, November 2014–April 2015. Participants were interviewed using a standardized questionnaire for demographic and clinical data.

### 2.2. Study Population

A total of 190 fecal samples were studied from two population groups (age 18–65 years): 102 from OPD visiting hospital from various parts of the country with the specimen representing gastrointestinal tract (stool) received for R/E + M/E (routine examination and microscopic examination) which was requested by the physician during general health checkup; also, a total of 88 (healthy population) out of which 58 students from college and 30 community people of Sandhikharka Municipality of Arghakhanchi District by door-to-door sampling were randomly selected in the study.

### 2.3. Specimen Collection and Processing

The methods for the collection, isolation, and identification were performed as described by American Society of Microbiology (ASM) [16] and analyzed accordingly. Stool samples containing Cary-Blair transport medium (culture swab transport system, Cary-Blair media, L-VV0052-2; Difco) were used and stored on ice pack and were transported to the lab within 3 days of collection. Fecal samples were inoculated on MacConkey agar (MAC) (HiMedia) plates. The plates were incubated at 37°C for 24 hr. All isolated colonies growing in the MAC agar were processed further for identification.

### 2.4. Inclusion Criteria

Patients aged between 18 and 65 years who showed willingness voluntarily to participate in the study with their written consent were enrolled for the study.

### 2.5. Exclusion Criteria

Patients were excluded from the study if they have had fever and diarrhoea. The specimen not fulfilling the criteria of ASM was also excluded from the study.

### 2.6. Identification of Bacterial Isolates

Identification of the isolates were done by the following standard microbiological techniques which involved morphological appearance of the colonies, Gram's staining reactions, catalase test, oxidase test, and other biochemical properties, for example, Sulphide Indole Motility (SIM) media, Simmons citrate media, Christensen's urea agar, Triple Sugar Iron agar (TSI), decarboxylase test media, Hugh and Leifson's OF (oxidative and fermentative) test media, MR/VP (methyl red/Voges Proskauer) broth, phenylalanine agar, nitrate reduction test [17], and others as required.

### 2.7. Phenotype Detection for ESBL and AmpC-*β*-Lactamase

The initial screening test for the production of ESBL was performed by using ceftazidime (CAZ) (30 *μ*g) and cefotaxime (CTX) (30 *μ*g) disks (Mast U.K.). If the zone of inhibition (ZOI) was ≤22 mm for ceftazidime and ≤27 mm for cefotaxime, the isolate was considered as a potential ESBL producer. The organism was swabbed on to a MHA (Mueller-Hinton agar) plate as done for the screening test in the antibiotic sensitivity test. Then, the combination disk method (CD) was applied for the confirmation of ESBL-producing strains. AmpC-*β*-lactamase production was detected by the 3-aminophenylboronic acid inhibitor-based detection method [18].

### 2.8. Combination Disk (CD) Method

CD methods were used for the confirmation of ESBL-producing strains in which CAZ and CTX alone and in combination with clavulanic acid (CA) (10 *μ*g) were used. An increased ZOI of ≥5 mm for either antimicrobial agent in combination with CA versus its zone when tested alone confirmed ESBL [19]. *E. coli* ATCC 25922 and *K. pneumoniae* ATCC 700603 were used as negative controls, respectively.

### 2.9. Test for AmpC-*β*-Lactamase Production

AmpC-*β*-lactamase enzyme production was detected by the 3-aminophenylboronic acid inhibitor-based detection method which was performed by inoculating on MHA and placing a disk containing 30 *μ*g of cefoxitin (HiMedia) and a disk containing 30 *μ*g of cefoxitin plus 400 *μ*g of boronic acid onto the agar. Inoculated plates were incubated overnight at 37°C. An organism that demonstrated a zone diameter around the disk containing cefoxitin and boronic acid that was 5 mm or greater than the zone diameter around the disk containing cefoxitin were considered an AmpC-*β*-lactamase producer [20].

### 2.10. Preparation of Disks Containing Boronic Acid

120 mg of phenylboronic acid (benzeneboronic acid; Sigma-Aldrich, Milwaukee, Wis.) was dissolved in 3 ml of dimethyl sulfoxide. Three milliliters of sterile distilled water was added to this solution. Twenty microliters of the stock solution was dispensed onto disks containing 30 *μ*g of cefoxitin. Disks were allowed to dry for 30 min and used immediately or stored in airtight vials with desiccant at 4°C and at −70°C [20].

### 2.11. Antibiotic Susceptibility Testing

The antimicrobial susceptibility tests were performed using the Kirby-Bauer disk diffusion method on Mueller-Hinton agar (HiMedia, India) as per CLSI recommendations [21]. The antibiotics tested in this study include amoxicillin (10 *μ*g), ceftazidime (30 *μ*g), cefotaxime (30 *μ*g), cefoxitin (30 *μ*g), cefepime (30 *μ*g), aztreonam (30 *μ*g), amoxicillin-clavulanate (30 *μ*g), piperacillin-tazobactam (100/10 *μ*g), gentamicin (10 *μ*g), imipenem (10 *μ*g), ciprofloxacin (5 *μ*g), and cotrimoxazole (25 *μ*g), respectively. All the antibiotics used were purchased from HiMedia Laboratories, Mumbai, India. Interpretation of antibiotic susceptibility results was made according to standard interpretative zone diameters suggested in CLSI guidelines [21]. In this study, if the isolates were resistant to at least three classes of first-line antimicrobial agents, they were regarded as MDR (multidrug resistance) [22].

### 2.12. Statistical Analysis

Descriptive data were generated. Continuous data were presented in Mean ± SD, and frequency table were generated for categorical variables. Chi-squared test and Fisher's exact test, where necessary, were performed for comparison of categorical variables. All the data were analyzed at 95% CI, and their corresponding *P* value < 0.05 was considered to be statistically significant. All data were entered, and statistics were performed using the SPSS version 20 software program.

### 2.13. Ethical Consideration

This study was approved by the Ethics Committee from Institutional Review Board of Institute of Medicine, TUTH. Written informed consent was obtained from each individual participating in the study.

## 3. Results

Demographics as well as clinical characteristics of the enrolled participants are presented in Table 1. The mean age of the enrolled OPD patients was 35.04 ± 14.23 years, where 55 (53.92%) were male. The mean age of the healthy population was 25.91 ± 11.93 years, where 46 (52.27%) were male. Of the 102 OPD patients, 47 (46.07%) participants had been hospitalized in the previous year and the proportions of antibiotic received within the three months prior to inclusion were 19 (18.62%), with 6 ciprofloxacin, 7 amoxicillin, 1 cefixime, 3 azithromicin, 1 ciprofloxacin + cotrimoxazole, and 1 amoxicillin + azithromicin. Similarly, among the healthy population, 14 (15.90%) patients had been hospitalized in the previous year and 8 (9.09%) participants received antibiotics prior to enrollment in the study: 1 subject received ciprofloxacin, 3 amoxicillin, 3 cefixime, and 1 azithromicin.

Of the total 190 participants (OPD + healthy population), a total of 260 isolates were identified; among these, *β*-lactamases (positive) were detected in 132 participants, with ESBL (+) 74/260 (28.46%), AmpC-*β*-lactamases (+) 35/260 (13.46%), and coproducers 23/260 (8.84% ), shown in Table 2. The proportion of ESBL, AmpC-*β*-lactamase, and coproducer producing *Enterobacteriaceae* in OPD participants was 47/152 (30.92%), 28/152 (18.4%), and 21/152 (13.81%), respectively. Also, the proportion of ESBL, AmpC-*β*-lactamases, and coproducer in healthy population was 27/108 (25%), 7/108 (6.4%), and 2/108 (1.8%), respectively.

Distribution of ESBL, AmpC-*β*-lactamase, and coproducer positive isolates in different species from hospital and community setting is shown in Table 3. For the outpatients, the most frequent ESBL-producing species was *E. coli* 33 (70.2%) followed by *K. pneumoniae* 6/47 (12.7%), *K. oxytoca* 3 (6.3%), *E. aerogenes* 2 (4.2%), and *C. freundii* 1 (2.1%), *C. koseri* 1 (2.1%), and *E. cloacae* 1 (2.1%). Among the coproducer, the most frequently detected isolates were *E.coli* 10/21 (47.6%), *C. koseri* 6/21 (28.6%), *K. pneumoniae* 2/21 (9.5%), *K. oxytoca* 1/21 (4.7%), and *E. aerogenes* 1/21 (4.7%). Similarly, among the AmpC-*β*-lactamase producer, *E. coli* were detected in half 14/28 (50.0%) of the isolates, followed by *K. pneumoniae* 5/28 (17.85%) and *C. freundii* 5/28 (17.85%), *E. aerogenes* 2/28 (7.8%), and *C. koseri* 1/28 (3.5%).

In healthy population, *E. coli* remained the most frequent ESBL producers 21/27 (77.8%) followed by *K. pneumoniae* 4/27 (14.21%) *and C. freundii* 2/27 (7.4%). Among the coproducer, *K. pneumoniae* and *C. freundii* were detected in 1/2 (50.0%) and 1/2 (50.0%), respectively. Also, among the AmpC producer, *K. pneumoniae* and *C. freundii* were detected in 5/7 (71.42%) and 2/7 (28.57%), respectively.

**Figure 1:**
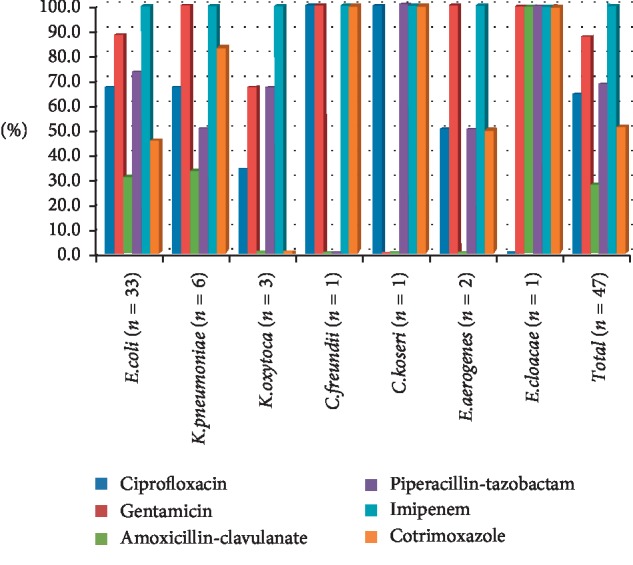
Antimicrobial susceptibility of ESBL-producing strains (*n* = 47) in OPD participants.

Antimicrobial susceptibility of ESBL-producing strains in OPD patients is illustrated in Figure 1. The overall antibiotics with the highest activity against the ESBL-producing isolates in OPD participants were imipenem 47 (100%) followed by gentamicin 41 (87.2%), piperacillin-tazobactam 32 (68.1%), ciprofloxacin 30 (63.8%), cotrimoxazole 24 (51.1%), and amoxicillin-clavulanate 13 (27.7%). Among the 33 isolates of *E. coli*, imipenem remained the most sensitive (100%), followed by gentamicin (87.9%) and piperacillin-tazobactam (72.7%). Of the total 6 *K. pneumoniae* isolates, imipenem and gentamicin were 100% sensitive to this pathogen, followed by cotrimoxazole (83.3%) and ciprofloxacin (66.7%). *K. oxytoca* appeared to be the most (100%) sensitive to imipenem, while gentamicin and piperacillin-tazobactam were 66.7% sensitive to this pathogen.

As far as *C. freundii* (*n* = 1) concerned, ciprofloxacin, gentamicin, imipenem, and cotrimoxazole were 100% sensitive to this isolate. With regard to *C. koseri* (*n* = 1), ciprofloxacin, piperacillin-tazobactam, imipenem, cotrimoxazole were most (100%) sensitive to this isolate, while gentamicin, amoxicillin-clavulanate remained 100% resistance. Among the isolates of *E. aerogenes* (*n* = 2), gentamicin and imipenem were 100% sensitive. Regarding *E. cloacae* (*n* = 1), gentamicin, piperacillin-tazobactam, imipenem, cotrimoxazole, and amoxicillin-clavulanate were 100% sensitive, while ciprofloxacin remained 100% resistant to this isolate.

**Figure 2:**
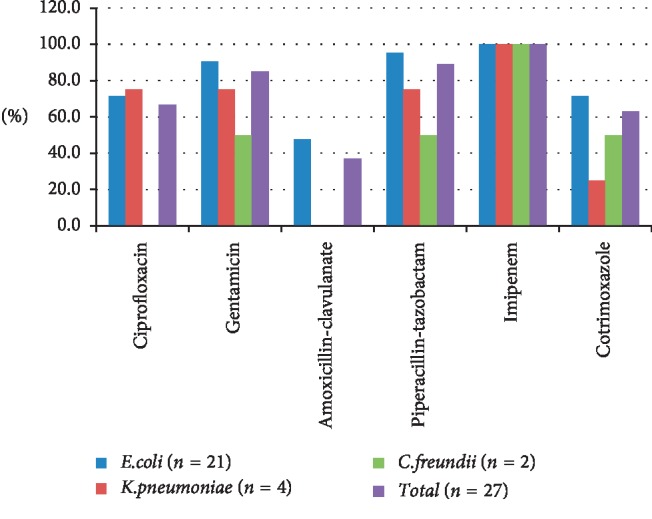
Antimicrobial susceptibility of ESBL-producing strains (*n* = 27) in healthy participants.

Antimicrobial susceptibility of ESBL-producing strains (*n* = 27) in healthy participants is illustrated in Figure 2. The antibiotics with the highest activity against the ESBL-producing isolates in healthy participants were imipenem 27 (100%), followed by piperacillin-tazobactam 24 (88.9%), gentamicin 23 (85.2%), ciprofloxacin 18 (66.7%), cotrimoxazole 17 (63.0%), and amoxicillin-clavulanate 10 (37.0%). *E. coli* was 100% sensitive towards imipenem, followed by piperacillin-tazobactam 20 (95.2%), gentamicin 19 (90.5%), ciprofloxacin and Cotrimoxazole 15 (71.4%), and amoxicillin-clavulanic acid 10 (47.6%). Similarly, imipenem was 100% sensitive towards *K. pneumoniae* followed by ciprofloxacin and gentamicin exhibiting 75% sensitive, whereas cotrimoxazole 25% and amoxicillin-clavulanic acid were 100% resistant to this pathogen. As far as *E. coli, K. pneumoniae*, *and C. freundii* concerned, imipenem remained 100% sensitive to these isolates.

Table 4 illustrates the association among various clinical characteristics with ESBL producers and nonproducers. For OPD patients, toilet facilities were significantly associated with the ESBL positive and negative carriage groups (*P* < 0.05). For the healthy populations, the sources of food intake were significantly associated with the ESBL positive and negative isolates (*P* < 0.05).

## 4. Discussion

New classes of enzymes causing resistant to *β*-lactam antibiotics have increasingly emerged over the last few decades. Among the various enzymes, ESBL remained the major that have spread worldwide and has been reported recently in fecal samples of outpatients as well as in healthy patients in many developed as well as developing countries [13, 23]. However, data on ESBL-producing *Enterobacteriaceae* in outpatients as well as in healthy population in Nepal are lacking.

In the present study, the overall fecal carriage of ESBL-producing *Enterobacteriaceae* (ESBL-E) in healthy and OPD patients was 28.46%, a slightly higher rate in outpatients (30.92%) compared with the healthy population (25%). This value is even higher than those reported in various parts of the regions, for instances, in our neighboring country India, 9.3% in hospitalized patients and 4.4% in healthy community subjects [13]; in Czech Republic, 8.2% in hospitalized patients and 3.2% in community subjects [23]; in Spain, 5.5% in outpatients and 3.7% in healthy volunteers [8]; in Netherlands, 10.1% in community patients [24]. However, recent studies have indicated a higher rate of ESBL-producing commensal enterobacterial strains in healthy population: (63.3%) from Egypt [25] and 58.2% from Thailand [15].

Thus, our finding indicates that ESBL-producing strains of bacteria are not only circulating in hospital environments but also in the community, and the clinicians need to be aware of these pathogens and they should deal with them accordingly.

There are several factors associated with the colonization and infection with ESBL producer: poor drug quality or inadequate posology, irrational use of antibiotics, unskilled practitioners, self-medication practice, unhygienic conditions accounting for the spread of resistant bacteria, and inadequate surveillance programs [26, 27]. The presence of ESBL-producing *E. coli* in the gut may result in the transfer of antibiotic-resistance determinants to other strains of *E. coli* and other organisms within the gastrointestinal tract [28]. Also, their presence increases the risk that other individuals will become carriers as a consequence of human-to-human transmission of resistant bacteria or through the environment [29].

In hospital setting, the prevalence of *E. coli* from various parts of the country reported was 29%–54.05% and prevalence of *K. pneumoniae* was 32.43%–61.3% [13, 23]. Also, in community setting, the prevalence of *E. coli* and *K. pneumoniae* varied: 66.7%–89.7% and 5.7%–24.0%, respectively [13, 15, 23, 27, 28]. Other strains reported in healthy population were *Enterobacter cloacae* (18.8%), *Citrobacter freundii* (10.4%), and *Klebsiella pneumoniae* (4.2%) [28].

In comparison, our study showed a high prevalence of *E. coli* (70.2%) in OPD population, whereas prevalence of *K. pneumoniae* (12.7%) in this study complies with the earlier studies. Prevalence of *E. coli* (77.8%) and *K. pneumoniae* (14.21%) demonstrated in our findings is consistent than that reported in the earlier studies. However, a lower proportion of *C. freundii* strain was observed in this healthy population (7.4%).

In contrast to ESBL enzymes, carriages of AmpC-*β*-lactamase positive bacterial enzymes have not been largely described. Also, to the best of the authors's knowledge, this is the first study that has been addressed the intestinal carriage of AmpC-*β*-lactamase in Nepalese setting, both in OPD as well as in healthy volunteer. Rashid et al. [13] identified the production of the AmpC-*β*-lactamase enzyme in 0.5% of bacterial isolates from the community and 1.7% in hospital isolates. Similarly, production of the AmpC-*β*-lactamase enzyme was detected in 1.1% of bacterial isolates from the community and in 0.3% hospital isolates in the Czech Republic [23]. In 2008, carriage of AmpC-*β*-lactamase in the gastrointestinal (GI) tract was detected in nearly 4% of healthy Danish army recruits. The same group was also demonstrated to carry ESBL-positive *Enterobacteriaceae* [30]. In the present study, the prevalence of AmpC-*β*-lactamase positive bacteria in the GI tract in OPD and healthy participants was 18.4% and 6.4%, respectively, and this value is even higher than those reported in the earlier studies.

In the present study, AmpC-*β*-lactamase-producing strain was dominantly found in *E. coli* and *K. pneumoniae* in OPD population, whereas in healthy subjects, AmpC-*β*-lactamase was found mostly in *K. pneumoniae* and *C. freundii*. Most importantly, the coproducer in this study was found to be (8.84%): a higher prevalence in OPD population (13.81%) compared with the healthy population (1.8%). It is crucial to note that 10 isolates of *Escherichia coli* and 6 isolates of *C. koseri* were detected in both ESBL and AmpC-*β*-lactamase in OPD population.

Several factors, such as human and animal antibiotic overuse, human cross-infection, and transmission from pets or other animals through the food chain, have contributed to the dissemination outside hospitals [30, 31]. In the present study, toilet facilities in OPD patients were significantly associated with the ESBL-producing *Enterobacteriaceae* (*P* < 0.03), while sources of food in healthy participants were significantly linked to ESBL-producing *Enterobacteriaceae* (*P*=0.003).

In this study, ESBL-producing *Enterobacteriaceae* in outpatients showed high resistance rates to cotrimoxazole (48.9%), ciprofloxacin (36.2%), and gentamicin (12.8%), but none of the isolates were resistant to imipenem. Similar results have been found in Cameroon from outpatients [32] and in other countries: Benin [33], Tanzania [34], and England [35].

Similarly, in healthy population, imipenem demonstrated 100% sensitive to these isolates; this value is similar to that demonstrated in previous studies [14, 25, 28]. Resistant to ciprofloxacin (33.3%) and gentamicin (14.8%) reported in this study was lower than those shown in previous studies [14]; however, resistant to piperacillin-tazobactam observed in this study was higher compared with earlier study (11.1% vs. 3.2%) [14]. Most importantly, these isolates in our study revealed a high resistance rate to amoxicillin-clavulanate (67%) and cotrimoxazole (37%).

In the present study, *Escherichia coli* remained the most common species isolated from both outpatients and healthy volunteers. In OPD population, ESBL-producing *E. coli* showed high resistance rates towards gentamicin (12.1%), which is lower than that reported in Nigeria (80–87%) [36] and in Madagascar (73.3% to 94.7%) [37], ciprofloxacin 33.4%, which is consistent than that reported in Egypt 39.4% [25]. Piperacillin-tazobactam (27.7%) showed high resistance towards *E. coli*. In healthy population, *E. coli* was 100% sensitive towards imipenem, which compiles the results shown in earlier studies [26, 28]. Also, the resistance of *E. coli* towards piperacillin-tazobactam (4.85%) and gentamicin (9.5%) observed in this study was lower than that reported in previous study [28].

Similarly, in this OPD population, imipenem and gentamicin reported to be 100% sensitive towards *K. pneumoniae*. In agreement with this finding, imipenem showed sensitive to all strains of ESBL-E [25, 37]; however, 18.9% amikacin was resistant to these isolates [25]. Likewise, ciprofloxacin in this study demonstrated to be 33.4% resistance to this pathogen which is in agreement with the previous report [25].

In agreement with the earlier study in healthy subjects [25], imipenem in our study demonstrated 100% sensitive towards *K. pneumoniae*. Ciprofloxacin and gentamicin exhibited 25% resistance towards *K. pneumoniae*, whereas cotrimoxazole exhibited 75% and amoxicillin-clavulanic acid exhibited 100% resistance to this pathogen. In comparison, resistant to *K. pneumoniae* in epidemiologic studies [28] is frequently seen in gentamicin (100%), ciprofloxacin (67%), and trimethoprim/ sulfamethoxazole (100%).

### 4.1. Limitation

We acknowledge that our study has been accomplished with some limitations. In this study, the patients who received antibiotics within 3 months were also included for analysis, which might have affected true picture of drug resistance.

## 5. Conclusion

Our study revealed a high prevalence of ESBL- and AmpC-*β*-lactamase-producing *Enterobacteriaceae* in the GI tract of both OPD and healthy subjects. The most prevalent ESBL producer strain appeared to be *E. coli* followed by *K. pneumoniae* in OPD as well as in healthy population. However, in healthy subjects, AmpC-*β*-lactamase-producing *K. pneumoniae* followed by *C. freundii* remained the most prevalent strain. Toilet use and food intake significantly associated with ESBL-E. Carbapenem remained highly (100%) sensitive to all strains of ESBL-E. While gentamicin, piperacillin-tazobactam, and fluoroquinolones demonstrated some rate of resistant, cotrimoxazole, amoxicillin-clavulanic acid shown to have high rate of drug resistance to ESBL-E both in OPD as well as in healthy population.

While colonization of the gastrointestinal tract by ESBL- and AmpC-*β*-lactamase-producing enteric pathogen increases the risk of infection, there is also a possibility that mobile carriers' resistance genes will be spread into a broad community. The drugs such as amoxicillin, ciprofloxacin, and sulfamethoxazole/ trimethoprim are the most popular drugs indicated for the treatment of most of the outpatients. Also, the presence of ESBL-producing strain may complicate the selection of antibiotics while considering the empirical therapy. In addition, gentamicin and piperacillin + tazobactam remained popular drugs for the treatment of the patients infected with ESBL–E and their increased resistant rate may bring the serious concern while treating these populations in the developing country like Nepal where the treatment option is limited. Thus, there remained a great importance on proper detection and control of these isolates both in OPD and community reservoir.

## Figures and Tables

**Table 1:** Characteristics of the study population.

Characteristics	OPD^*∗∗*^ patients *N* = 102	Healthy population^*∗*^*N* = 88
Age, mean ± SD	35.04 ± 14.23	25.91 ± 11.93
Sex		
Male	55 (53.92)	46 (52.27)
Female	47 (46.07)	42 (47.72)
Hospitalization (in previous years)		
Yes	47 (46.07)	14 (15.90)
No	55 (53.92)	74 (84.09)
Antibiotic use <3 months		
Yes	19 (18.62)	8 (9.09)
No	68 (66.7)	68 (77.72)
Unknown	15 (14.70)	12 (13.63)
Antibiotics used		
Ciprofloxacin	6	1
Amoxicillin	7	3
Cefixime	1	3
Azithromicin	3	1
Ciprofloxacin + cotrimoxazole	1	0
Amoxicillin + azithromicin	1	0

^*∗*^Healthy population (*N* = 88) = 58 students of college +30 community participants. ^*∗∗*^OPD (outpatient department).

**Table 2:** Distribution of *β*-lactamases in different clusters of sample.

	Total (OPD + healthy population)	OPD patients (total isolates, *N* = 152)	Healthy population (total isolates, *N* = 108)
ESBL	74/260 (28.46%)	47/152 (30.92%)	27/108 (25%)
AmpC-*β*-lactamase	35/260 (13.46%)	28/152 (18.4%)	7/108 (6.4%)
Coproducer	23/260 (8.84%)	21/152 (13.81%)	2/108 (1.8%)
Total	132	96	36

**Table 3:** ESBL, AmpC-*β*-lactamase, and coproducer positive isolates in different species from hospital and community setting.

Organisms	OPD population		Healthy population			
	ESBL+	Coproducer	AmpC+	ESBL+	Coproducer	AmpC+
*E. coli*	33/47	10/21	14/28	21/27	0	0
*N* = 161	(70.2)	(47.6)	(50.0)	(77.8)		
*K. pneumoniae*	6/47	2/21	5/28	4/27	1/2	5/7
*N* = 50	(12.7)	(9.5)	(17.85)	(14.21)	(50.0)	(71.42)
C. *freundii*	1/47	1/21	5/28	2/27	1/2	2/7 (28.57)
*N* = 14	(2.1)	(4.7)	(17.85)	(7.4)	(50.0)	
*K. oxytoca*	3/47	1/21	1/28		0	0
*N* = 19	(6.3)	(4.7)	(3.5)			
C. *koseri*	1/47	6/21	1/28	0	0	0
*N* = 6	(2.1)	(28.6)	(3.5)			
*E. aerogenes*	2/47	1/21	2/28			
*N* = 5	(4.2)	(4.7)	(7.8)	0	0	0
*E. cloacae*	1 (2.1)	0 (0.00)	0 (0.00)	0	0	0
*N* = 2						
Total	*N* = 47	*N* = 21	*N* = 28	*N* = 27	*N* = 2	*N* = 7

**Table 4:** Association among various clinical characteristics with ESBL producers and nonproducers.

Covariate	OPD (outpatient department)		*P* ^*∗*^	Healthy population		*P* ^*∗*^
	ESBL (+)	ESBL (−)		ESBL (+)	ESBL (−)	
Age, mean ± SD	35.4 ± 13.4	34.8 ± 14.7	.848	25.7 ± 10.7	25.9 ± 12.3	0.921
Gender			0.56			0.73
Male	27	55		12	39	
Female	20	50		15	42	
Antibiotic use <3 months (+)	7	19	0.63	7	13	0.25
ABX use with /without prescription			0.66			0.21
With prescription	34	75		22	68	
Without prescription	11	28		4	13	
Both	2	2		1	0	
Hospitalization previous year (+)	21	45	0.83	5	10	0.42
Previous worked in hospital (+)	2	8	0.43	9	16	0.14
Visit hospital within 3 months (+)	20	51	0.49	19	44	0.14
Smoking history (+)	11	12	0.057			
Alcohol intake (+)	13	22	0.36	6	8	0.09
Toilet facilities (+)	45	105	0.033	25	80	0.091
Meat consumption (+)	40	93	0.55	25	70	0.39
Milk consumption	46	100	0.44	26	81	0.082
Source of food intake			0.16			0.003
Home	41	77		19	72	
Hotel	1	4		0	4	
Both	5	24		8	5	
Travel abroad (+)	10	31	0.29	5	5	0.055

^*∗*^Pearson chi-squared test was applied, where a *P* value ≤0.05 was considered to be statistically significant.

## Data Availability

All data generated or analyzed during this study are included in this published article.

## Abbreviations

AMC: Amoxicillin-clavulanic acid
ABX: Antibiotics
ASM: American Society for Microbiology
ATCC: American Type Culture Collection
BA: Blood agar
CAZ: Ceftazidime
CTX: Cefotaxime
CD: Combination disk
OPD: Outpatient department
R/E + M/E: Routine examination + Microscopic examination
CLSI: Clinical and Laboratory Standards Institute
ESBL-E: Extended-spectrum *β*-lactamase-producing *Enterobacteriaceae*
CRO: Ceftriaxone
D/W: Distilled water
ESBL: Extended-spectrum *β*-lactamase
Fig: Figure
gm: Gram
Hrs: Hours
K/A: Alkaline/acid
MA: MacConkey agar
MDR: Multidrug resistant
MHA: Mueller-Hinton agar
mm: Millimeter
No.: Number
SIM: Sulphide indole motility
TSI: Triple Sugar Iron
TUTH: Tribhuvan University Teaching Hospital
WHO: World Health Organization
*μ*g: Microgram
